# Fumonisin and ochratoxin-producing strains of *Aspergillus* section *Nigri* are associated with onion (*Allium cepa* L.) bulbs sold in markets in southwest Nigeria

**DOI:** 10.3389/ffunb.2025.1563824

**Published:** 2025-03-24

**Authors:** Catherine Oluwakemi Esuola, Alejandro Ortega-Beltran

**Affiliations:** ^1^ Biotechnology Research Unit, National Horticultural Research Institute, Ibadan, Oyo-State, Nigeria; ^2^ Pathology and Mycotoxin Unit, International Institute of Tropical Agriculture, Ibadan, Oyo-State, Nigeria

**Keywords:** mycotoxigenic *Aspergillus niger*, fumonisin B_2_ (FB_2_), ochratoxin (OTA), food security, multiplex PCR (polymerase chain reaction), postharvest losses, onion bulbs

## Abstract

**Introduction:**

Onion bulbs are edible, nutritious vegetables and spices. In Nigeria, mass propagation of onion seedlings is limited due to infection of the onion bulbs by *Aspergillus* section *Nigri*, especially *Aspergillus niger* strains. Mycotoxin-producing *A. niger* strains are detrimental to public health. Hence, this study was undertaken to screen the locally sourced onion bulbs for fumonisin B_2_ (FB_2_) [Multiplex A: *fum6* (374 bp), *fum8* (272 bp), *fum13* (168 bp), and *fum19* (479 bp) and Multiplex B: *fum1* (452 bp), *fum7* (238 bp), *fum3* (173 bp), and *fum14* (321 bp)] and ochratoxin A [OTA; *pks15ks* (776 bp)] *A. niger* biosynthetic genes.

**Methods:**

Thus, 100 onion bulbs were collected from four different local markets (Dugbe, Agbowo, Sasa, and Omi). The surface of each bulb was disinfected, and after incubation, isolates of *A. niger* were detected. *A. niger* isolates were cultured on potato dextrose agar. Genomic DNA was extracted from isolates of *A. niger*. Multiplex PCR confirmed the presence and absence of FB_2_ (*fum*) and OTA (*pks15ks*) biosynthetic genes.

**Results and Discussion:**

A total of 200 isolates of *A. niger* were isolated from the onion bulbs, and 22 (11%) isolates amplified at least two *fum* genes, while three (1.5%) amplified the *pks15ks* gene. All isolates were positive for *fum1* and *fum19*. The highest/lowest percentage of the location/isolates of *fum* and *pks15ks* was Agbowo/Omi with 32.0%/2.1% and Dugbe/Agbowo with 6.7%/2.3%, respectively. Hence, the use of multiplex PCR to detect FB_2_ and OTA biosynthetic genes in the isolated *A. niger* strains from the locally sourced onion bulbs will assist onion growers in the mass production of healthy onion seedlings with export potential and quality. Early detection of FB_2_ and OTA biosynthetic genes is important to predict possible mycotoxin-producing *A*. section *Nigri* in onion bulbs.

## 1 Introduction

Onion (*Allium cepa* L.) is a nutritious vegetable and spice used worldwide, belonging to the Liliaceae family (Griffiths et al., 2002; Steentjes et al., 2021). It is widely grown for its edible bulbs, which have nutritional, medicinal, and therapeutic properties (Kim et al., 2021; Li et al., 2011; Sagar et al., 2022; Steentjes et al., 2021). Onions rank second after tomatoes among the various horticultural crops and vegetables humans consume (Fu et al., 2019; Griffiths et al., 2002). In Nigeria, onions are mainly produced by subsistence farmers in the north of the country, from where they are transported to local markets across the country (Dogon-Daji and Mohammad, 2022; Shehu and Muhammad, 2012). A major challenge to onion production is the high postharvest losses caused by the black mold, *Aspergillus niger*. This fungus belongs to *Aspergillus* section *Nigri* and leads to postharvest losses as a result of contamination of onion seeds, method and location of cultivation, environmental conditions, and/or storage conditions after harvest (Shehu and Muhammad, 2012; Steentjes et al., 2021).

Another challenge affecting onion production in Nigeria is the limited availability of planting materials. Onion seeds are expensive, leading to the current high cost of onion production in Nigeria. Micropropagation of onion seedlings using shoot-tip explants can generate clean and uniform onion plants. Unfortunately, the shoot-tip explants are prone to fungal contaminants, hindering their growth. *A*. *niger* strains cause black mold rot disease when producing *in vitro* shoot tips. Strains of *A*. *niger* are known to produce the mycotoxins fumonisin B_2_ (FB_2_) and ochratoxin A (OTA). These mycotoxins endanger human and animal health upon high, repeated exposures (El-Dawy et al., 2024; Frisvad et al., 2011). Mycotoxin contamination limits the export potential and quality of food products.

Multiplex PCR primer sets were developed by Palumbo et al. (2013) to detect the genetic basis for the loss of FB_2_ biosynthetic genes, *fum*, in FB_2_^-^ nonproducing (FB_2_^-^) *A. niger* and *A. awamori* strains. Multiplex PCR primer sets have been used to detect *fum* genes in *A. welwitschiae* strains isolated from onions (Gherbawy et al., 2015). Interestingly, *A. niger* strains lacking *fum* genes could translate as useful ecological or industrial fungi species (Palumbo et al., 2013). Mycotoxin production by *A*. section *Nigri* has also been reported in other *Allium* sp. such as garlic (*A*. *sativum* L.) (Anjorin et al., 2021; Vanzela et al., 2020; Zakaria, 2024).

Other food products reported to be contaminated with FB_2_ include grapes, dried fruit, Brazil nuts, coffee beans, cocoa, and maize (Ferrara et al., 2020; Massi et al., 2016a). Primarily, FB_2_ production in maize grains is by *Fusarium verticilloides* (Ding et al., 2024; Logrieco et al., 2020), but it has also been produced by *A*. *niger* in maize kernels from the United States (69%) and Italy (38%) (Susca et al., 2014). Industrial strains of *A. niger* used for producing citric acid have been reported to be toxigenic strains on media (Frisvad et al., 2007). Therefore, strains of *A. niger* with inactivated gene clusters for FB_2_ and OTA are recommended for biotechnological and industrial fermentation, and food and beverage applications (Frisvad et al., 2011).

OTA causes nephrotoxic effects in mammalian species; it is the most toxigenic and common ochratoxin found in food in several countries (Massi et al., 2016a; Sartori et al., 2010). OTA producers have been reported in *A. niger* strains from onions, grapes, and other foods (Bucheli and Taniwaki, 2002; Massi et al., 2016a). A polyketide synthase gene encodes for the production of OTA (O’Callaghan et al., 2003). The expression of the key mycotoxin biosynthetic genes is useful as a Hazard Analysis and Critical Control Point (HACCP) (Niessen, 2008). A better understanding of the mycotoxin biosynthetic genes will aid in the early prediction of mycotoxin production in food samples (Sartori et al., 2010).

Research on micropropagation methods to increase the current production of quality and disease-free onion seedlings all year round is ongoing at the National Horticultural Research Institute, Ibadan, Nigeria. However, the *in vitro* onion bulb shoot-tip explants are contaminated with pathogenic fungi, mostly *A*. *niger*. Additionally, it is unclear whether these *in vitro* contaminated shoot-tip explants from locally sourced onion bulbs in the Ibadan metropolis are infected with mycotoxin-producing *A*. section *Nigri*. Therefore, this study aims to screen onion bulbs from four different local markets in the Ibadan metropolis for possible contamination with fumonisin and OTA-producing *A*. section *Nigri* using FB_2_ multiplex PCR biosynthetic gene primer sets (A and B) and the OTA biosynthetic gene primer PK15KS.

## 2 Materials and methods

### 2.1 Collection of onion bulb samples and study area

In January 2022, during the dry season period (onion bulbs typically contain *Aspergillus* propagules throughout the year), onion bulbs were purchased from five different sellers (five whole onion bulbs per seller) from four different local markets in Ibadan, Southwest Nigeria. These markets are located in Agbowo (7°44′ N, 3°91′ E), Dugbe (7°39′ N, 3°87′ E), Omi-Adio (7°39′ N, 3°78′ E), and Sasa (7°48′ N, 3°91′ E). Therefore, 100 bulbs were collected and taken to the Pathology and Mycotoxin Unit, International Institute of Tropical Agriculture, Ibadan for further analysis.

### 2.2 Media preparation

Potato dextrose agar (PDA; Sigma Aldrich, 32 g L^-1^) was prepared using distilled water and sterilized at 121°C for 20 min. Lactic acid (0.5%) was added to the PDA media to prevent infection with bacterial contaminants before dispensing into 9 mm Petri plates.

### 2.3 Preparation and incubation of onion bulbs for fungal isolation

Twelve crisper boxes, each approximately 10 × 5 × 4 cm, were lined with six sterile paper towels separately and moistened with 100 mL sterile distilled water each. Onion bulb samples were cut longitudinally into halves. They were then surface sterilized by submersion in 70% ethanol for 3 min, followed by submersion in 3.5% sodium hypochlorite (Reckitt^®^) [v/v] for 5 min. Thereafter, the bulbs were rinsed with sterile distilled water three times. Onion bulbs with the cut surface up were placed on sterile paper towels in the crisper boxes. Each box contained 15 bulbs per location. The boxes were sealed with cling film and placed on a clean surface at room temperature and observed for fungal growth and the appearance of black mold *A*. *niger* strains until the eighth day. Colonies of *A. niger* were aseptically picked from infected bulbs and transferred to fresh PDA plates, sealed with parafilm, and incubated for 5 days at 30°C. Serial dilutions of the isolates were made to obtain single spores. The single spores were inoculated on PDA and the mycelia were harvested for total genomic DNA extraction and further analysis. The mycelia were also kept in 50% glycerol at -80°C for long-term storage.

### 2.4 Genomic DNA extraction

The mycelia of the isolates of *A. niger* were collected for DNA extraction according to the methods of Callicott and Cotty (2015) and Sambrook and Russell (2001). Briefly, spores from pure cultures of 5-day-old cultures of *A. niger* grown on PDA were harvested by adding 1.5 mL of 0.1% TWEEN^®^80 to the culture. Then, 1.2 mL of the suspension was aseptically transferred to a sterile 1.5 mL Eppendorf tube. The suspension was centrifuged at 8,000× g for 5 min. The supernatant was carefully removed without disturbing the precipitate. Lysis buffer (450 μL: 270 mM Tris, 90 mM EDTA, 1% SDS, pH 8.0) was added to each tube and vortexed briefly to resuspend the precipitate. Eppendorf tubes were placed in an Eppendorf ThermoMixer^®^ at 60°C and 8,000× g for 60 min, and thereafter, centrifuged at 14,000× g for 30 min. Then, 340 μL of the supernatant was transferred to a newly labeled sterile Eppendorf tube, and 340 μL of refrigerated 4 M ammonium acetate was added. The suspension was thoroughly mixed. Afterward, 680 μL ice-cold absolute ethanol was added, the content was thoroughly mixed, and the tube was placed in the freezer at -20°C for 1 hr. The resulting mixture was then centrifuged at 14,000× g for 5 min, the supernatant was carefully removed, and the pellet was washed with ice-cold 70% ethanol at 14,000× g for 5 min. The Eppendorf tubes were left to dry for 90 min and the pellet was resuspended in 50 μL sterile nuclease-free water and gently mixed. The DNA concentration was determined using a Nanodrop spectrophotometer (Thermo Fisher Scientific™) and stored at -20°C for further analysis.

### 2.5 PCR conditions and gel electrophoresis

The Multiplex primer sets A and B used for the multiplex PCR fumonisin (Palumbo et al., 2013) and ochratoxin (Ferracin et al. 2012) analyses are described in **Table 1** (oligonucleotide primers from Inqaba Biotec™). Multiplex PCR analyses were used for the fumonisin primers according to Palumbo et al. (2013). The primers were grouped into Multiplex A: *fum6* (374 bp), *fum8* (272 bp), *fum13* (168 bp), and *fum19* (479 bp); and Multiplex B: *fum1* (452 bp), *fum7* (238 bp), *fum3* (173 bp), and *fum14* (321 bp). The fumonisin fragment of each region was amplified using the following reagent concentrations: 2.2 μL OneTaq Quick-Load New England, BioLabs 5X Master Mix, 0.22 μL, 10 μM of each primer, 2 μL 10 ng/μL template DNA, and 6.36 μL nuclease-free water for a final volume of 11 μL. Initial denaturation was at 95°C for 1 min, followed by 35 cycles of 95°C for 20 s, annealing at 60°C for 1 min, and extension at 68°C for 2 min, with a final extension step at 68°C for 5 min.

**Table 1:** Fumonisin B_2_ (FB_2_) and ochratoxin A (OTA) biosynthetic genes, sequences, and expected amplicon size of the primer pairs used in this study, according to Palumbo et al. (2013) and Ferracin et al. (2012).

	Gene	Primer	Sequence (5’–3’)	Base pairs
Multiplex A	*fum6*	fum6F.4pl	GAAATGGGCGCGTCTTGGGGAA	374
		fum6R.4pl	CGCTCAACCGCTCTCCCGTTTT	
	*fum8*	fum8F.4pl	CCGGGACTTGAAAGCATGGCGT	272
		fum8R.4pl	TGACAACCTCTCGTGTCGGGCA	
	*fum13*	fum13F.4pl	TGCGCCAACTGTCCAAGGAACC	168
		fum13R.4pl	TGGCGGTGGGTTGTCGAAATGG	
	*fum19*	fum19F.4pl	TAGATGGCGAGTTCGGGTGGCA	479
		fum19R.4pl	TTCGGTATCAGCGTCGAGGCCA	
Multiplex B	*fum1*	fum1F.4pl	TGGCGATTGGTCGTCCAGGTCT	452
		fum1R.4pl	GCCACCGATGTCCACAAGCGAA	
	*fum7*	fum7F.4pl	TACAGACGGCGAACGCTCCAGA	238
		fum7R.4pl	GCCTTCAGCAACGCCTCCTGTT	
	*fum3*	fum9F.4pl	AGAACCGCAGACCCTTCACCGT	173
		fum9R.4pl	CACGCTCACTGAACGCCCACTT	
	*fum14*	fum14F.4pl	TTTGGGCTGTGTCGGCATGGTC	321
		fum14R.4pl	ACGCCGTGTAACCATTCGCCAG	
OTA	An15g07920 (*pks15ks*)	PKS15KS-F	F-CAATGCCGTCCAACCGTATG	776
		PKS15KS-R	R-CCTTCGCCTCGCCCGTAG	

The ochratoxin A fragment was amplified using the following reagents: 5.5 μL of OneTaq Quick-Load New England, BioLabs 2X Master Mix, 0.22 μL 20 μM of each primer (PK15KS, **Table 1**), 2 μL 10 ng/μL template DNA, and 3.06 μL nuclease-free water to make a final volume of 11 μL. The PCR conditions of the Susca et al. (2016) study were used, with an initial denaturation at 95°C for 2 min, followed by 35 cycles of 94°C for 30 s, annealing at 47°C for 30 s, and extension at 72°C for 30 s, with a final extension step at 72°C for 7 min.

The amplified fragments were separated on a 1% agarose gel, which contained 1× Tris-acetate-EDTA (TAE) buffer pre-stained with 5% loading dye (SafeView™) before being poured into the electrophoresis tray and then placed in the tank containing 1× TAE for electrophoresis at 90 min, 110 V. The amplified band patterns were viewed with the gel documentation system.

### 2.6 Pathogenicity test

The 22 A. *niger* isolates that tested positive for the FB_2_ and OTA biosynthetic genes were used for the pathogenicity test. The isolates were cultured on PDA, and 5 days after, spores were picked from each plate and inoculated into sterilized and fungicide-treated onion bulbs in the crisper boxes, and their pathogenic effects were observed at 8 days after inoculation (DAI). Sterile water inoculated into sterile onion bulbs served as the control. The experiment was performed with three biological replicates (n=3).

## 3 Results

### 3.1 *A. niger* strains isolated from the onion bulbs

**Figure 1:**
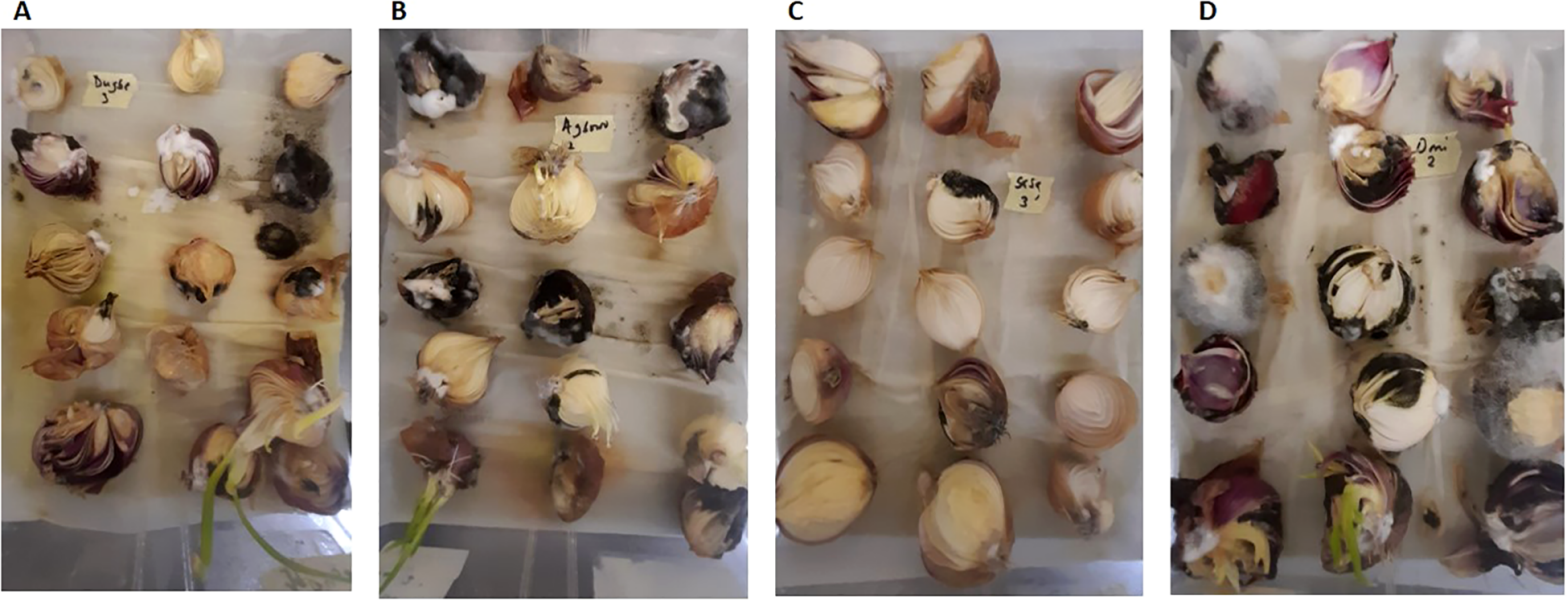
*Aspergillus niger* strains isolated from the onion bulbs per location in different crisper boxes at 8 days after inoculation (DAI). **(A)** Onion bulbs from Dugbe market. **(B)** Onion bulbs from Agbowo market. **(C)** Onion bulbs from Sasa market. **(D)** Onion bulbs from Omi market.

**Figure 2:**
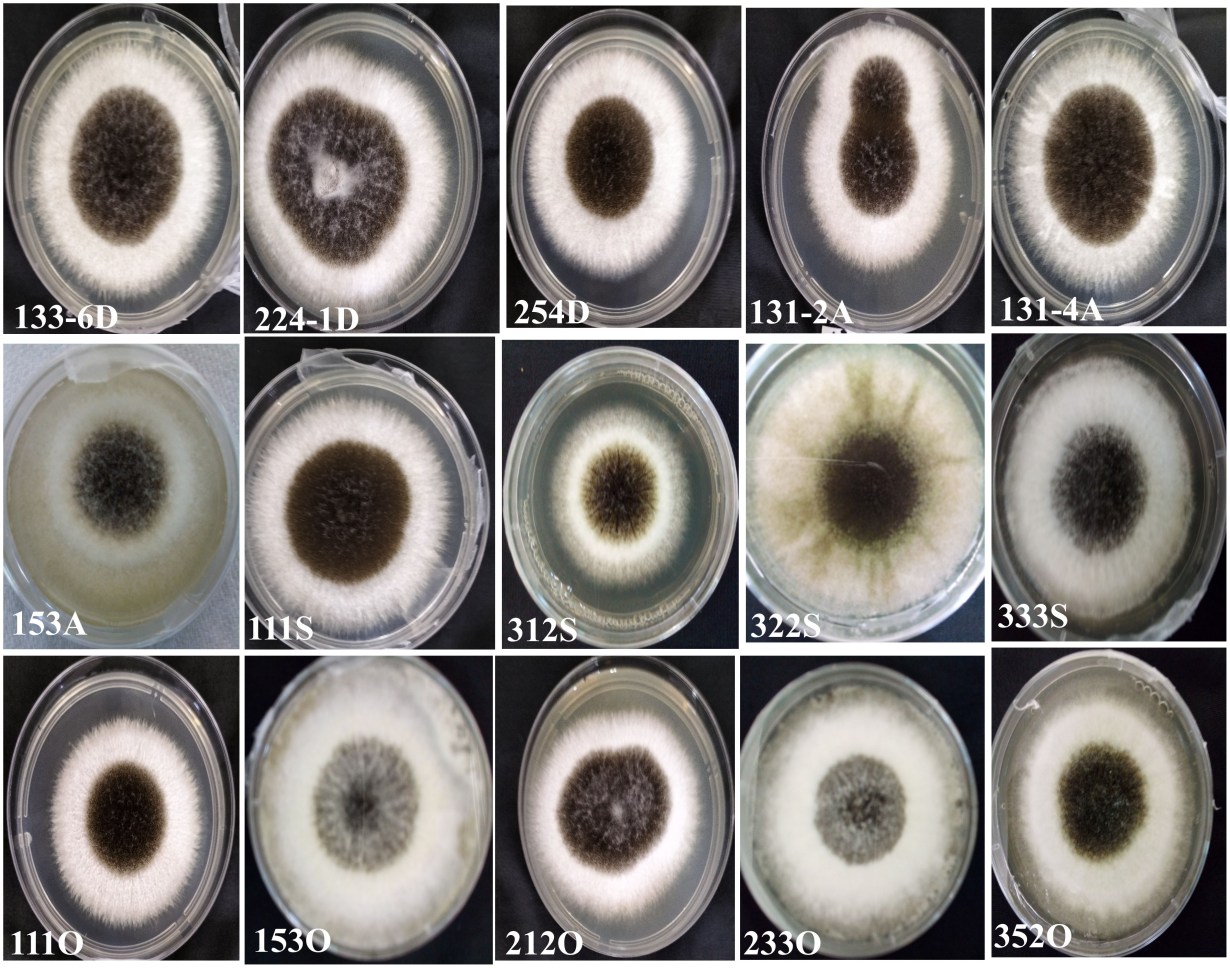
Some *Aspergillus niger* strains from the black mold-infected onion bulbs in the crisper boxes and cultured on potato dextrose agar (PDA) at 5 days after inoculation (DAI).

There was an appearance of black mold (*A*. *niger*) causing rot of the onion bulbs at 8 DAI in the crisper boxes (**Figure 1**). Sections of fungal growth were transferred to PDA plates. The different strains of *A*. *niger* were coded according to seller number, box number, bulb number, and location (**Figure 2**). A total of 200 isolates were obtained: 30 from Dugbe, 44 from Agbowo, 30 from Sasa, and 96 from Omi markets (**Table 2**).

**Table 2:** Number of *A. niger* isolates per location and the percentage that tested positive for FB_2_ and OTA biosynthetic genes.

Location	No. of *A. niger* isolates	% positive for FB_2_	% positive for OTA
Dugbe	30	16.7	6.7
Agbowo	44	32.0	2.3
Sasa	30	3.3	0.0
Omi	96	2.1	0.0
Total	200	11	1.5

### 3.2 FB_2_ biosynthetic genes in the *A. niger* isolates

**Figure 3:**
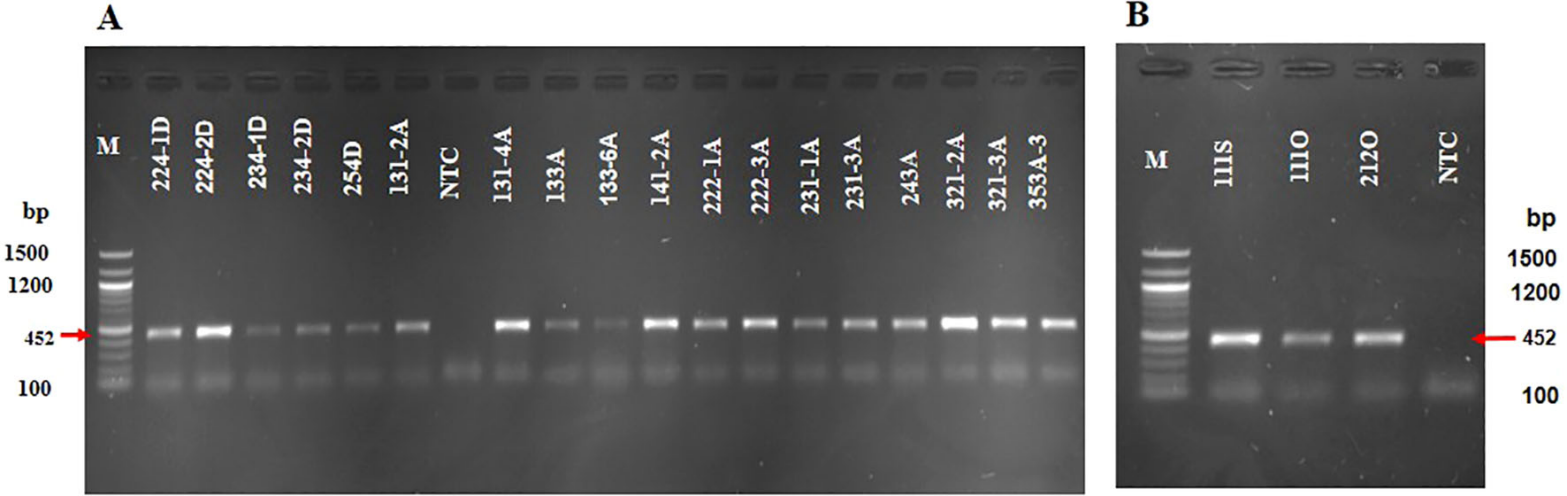
Agarose gel showing the fumonisin biosynthetic gene *fum1* (452 bp) detected in the *A niger* isolates. **(A)** Lanes 1–20: M (100 bp marker), 224-1D, 224-2D, 234-2D, 234-3D, 254D, 131-2A, NTC (no template control), 131- 4A, 133A, 133-6A, 141-2A, 222-1A, 222-2A, 231-1A, 231-3A, 243-2A, 251A, 321-2A, 321-3A, and 353-3A. **(B)** Lanes 1–5: M (100 bp marker), 111S, 111O, 212O, and NTC.

**Figure 4:**
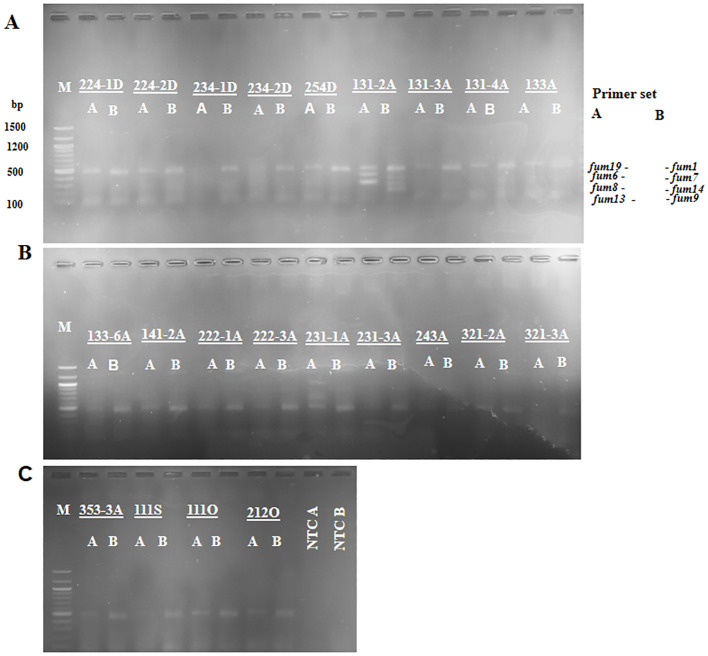
Agarose gel showing the multiplex PCR, primer sets A, and primer sets B of fumonisin biosynthetic genes detected in some of the *A niger* isolates. **(A)** Lanes 1–19: M (100 bp marker), 224-1D, 224-2D, 234-2D, 234-3D, 254D, 131-2A, 131-3A, 131-4A, and 133A. **(B)** Lanes 1-19: M (100 bp marker), 133-6A, 141-2A, 222-1A, 222-2A, 231-1A, 231-3A, 243A, 251A, 321-2A, and 321-3A. **(C)** Lanes 1–11: M (100 bp marker), 353-3A, 111S, 111O, 212O, NTC A (no template control primer set A), and NTC B (no template control primer set B).

There were 22 A*. niger* isolates positive for FB_2_ biosynthetic genes: 5 (16.7%), 14 (32.0%), 1 (3.3%), and 2 (2.1%) from Dugbe, Agbowo, Sasa, and Omi markets, respectively (**Figures 3A, B**, **4A–C**; **Tables 2**, **3**; also see **Supplementary Figures S1**–**S5**). The percentage calculation is given below. Isolate 131-2A was positive for *fum* genes in the primer sets A and B, except for *fum13* (**Figure 4A**; **Table 3**).

**Table 3:** Presence (+) and absence (−) of the fumonisin B_2_ (FB_2_) and ochratoxin A (OTA) biosynthetic genes in the *A. niger* isolates.

Location	Isolate	Multiplex A				Multiplex B				OTA
		*fum6*	*fum8*	*fum13*	*fum19*	*fum1*	*fum7*	*fum3*	*fum14*	*pks15ks*
DUGBE	224-1D	–	–	–	+	+	–	–	–	–
	224-2D	–	–	–	+	+	–	–	–	+
	234-1D	–	–	–	+	+	–	–	–	–
	234-2D	–	–	–	+	+	–	–	–	+
	254D	–	–	–	+	+	–	–	–	–
AGBOWO	131-2A	+	+	–	+	+	+	+	+	+
	131-3A	–	–	–	+	+	–	–	–	–
	131-4A	–	–	–	+	+	–	–	–	–
	133A	–	–	–	+	+	–	–	–	–
	133-6A	–	–	–	+	+	–	–	–	–
	141-2A	–	–	–	+	+	–	–	–	–
	222-1A	–	–	–	+	+	–	–	–	–
	222-3A	–	–	–	+	+	–	–	–	–
	231-1A	–	–	–	+	+	–	–	–	–
	231-3A	–	–	–	+	+	–	–	–	–
	243A	–	–	–	+	+	–	–	–	–
	321-2A	–	–	–	+	+	–	–	–	–
	321-3A	–	–	–	+	+	–	–	–	–
	353-3A	–	–	–	+	+	–	–	–	–
SASA	111S	–	–	–	+	+	–	–	–	–
OMI	111O	–	–	–	+	+	–	–	–	–
	212O	–	–	–	+	+	–	–	–	–


$$\text{Percentage calculation}=\frac{\text{Total number of positive isolates}}{\text{Total number of isolates/location}}\times 100$$

### 3.3 OTA biosynthetic gene detected in the *A. niger* isolates

**Figure 5:**
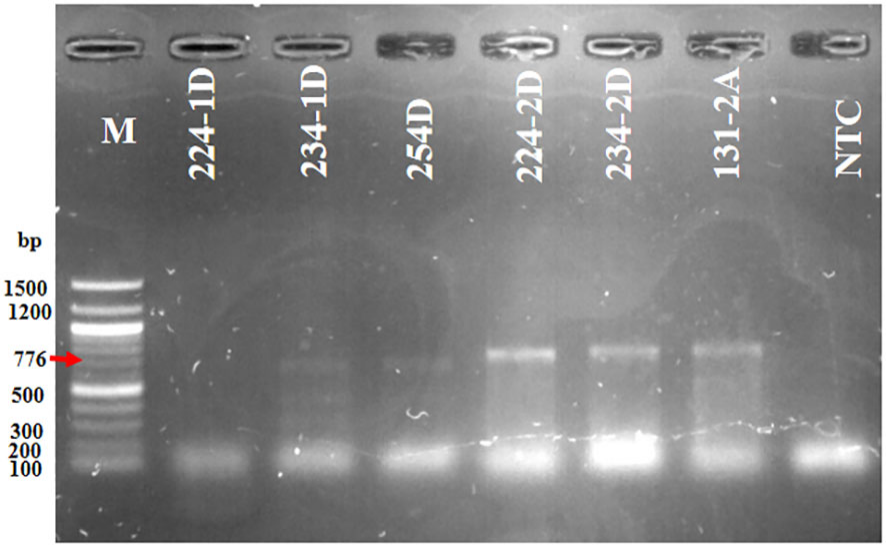
Agarose gel showing the ochratoxin (OTA) biosynthetic gene *pks15ks* (776 bp) detected in some of the *A. niger* isolates. Lane 1: M (100 bp marker); lanes 2–4: 224-1D, 234-1D, and 254D are negative; lanes 5–7: 224-2D, 234-2D, and 131-2A are positive for the *pks15ks* (776 bp) gene; and lane 8: NTC (no template control).

*A. niger* isolates 224-2D, 234-2D, and 131-2A were positive for the OTA biosynthetic gene *pks15ks* (776 bp) (**Figure 5**; **Table 1**). The result indicates that 6.7% of *A. niger* isolates from Dugbe and 2.3% from Agbowo were detected to have OTA, whereas none were detected from Sasa and Omi (**Figure 5**; **Supplementary Figure S6**; **Table 2**).

### 3.4 Pathogenicity test

All the *A. niger* isolates tested for their pathogenicity in the sterilized onion bulbs were able to cause black rot disease at 8 DAI, whereas no black rot disease was observed in the control onion bulbs inoculated only with sterile water.

## 4 Discussion

Onion production in Nigeria is limited by conventional seed materials for commercial propagation. The onion seeds are mostly susceptible to *A. niger* infection, which is more pronounced during post-harvest storage, leading to drastic loss of onion bulbs for consumption and export. Here, we studied onion bulbs collected from local markets in the Ibadan metropolis for possible contamination with mycotoxin-producing *A*. section *Nigri*. Onion bulbs in Nigeria are mostly produced in the northern parts and transported to other areas of the country. These northern areas are usually hotter and may also be a spreading ground for mycotoxigenic *A. niger* strains as the tropics are regions in which the spread of mycotoxigenic fungal species is higher (Ortega-Beltran and Bandyopadhyay, 2023). The means of transportation and eventual storage of onion bulbs could also be a spreading point for *A. niger* black mold. In the various markets, onion bulbs were stored in open places such as baskets and plastic bags, which form moisture upon exposure to sunlight.

*A. niger* has the potential to produce two groups of carcinogenic mycotoxins, i.e., fumonisins and ochratoxins, in food and feed (Frisvad et al., 2011; Susca et al., 2016). A step forward in preventing mycotoxins such as fumonisin and ochratoxin is the use of multiplex PCR analysis to detect the FB_2_ and OTA promptly before the food products reach the consumer, as in the case of onions in this study. Fumonisin-producing *A*. section *Nigri* strains were detected by multiplex PCR analysis and the patterns of FB_2_ were useful to indicate the extent and ability of the tested strains to produce fumonisin. In their study, Palumbo et al. (2013) developed a multiplex PCR primer sets to amplify fragments of eight *A. niger* FB_2_ *fum* gene orthologs (**Table 1**) to help identify FB_2_^-^ strains for industrial, agricultural, and ecological purposes. They identified five different patterns of amplification of the tested *fum* genes from 47 FB_2_^-^ strains: pattern 1, where the *fum3*, *fum7*, *fum13*, and *fum14* were amplified; pattern 2, where only *fum1* amplicon was amplified; pattern 3, the most common pattern, where only *fum1* and *fum19* were amplified; pattern 4, where only *fum6* amplicon was not amplified; and pattern 5, where all FB_2_ *fum* genes were amplified. Interestingly, they confirmed that these patterns were able to separate the FB_2_^-^ strains into two different species: patterns 2 and 3 were *A. awamori*, whereas patterns 1, 4, and 5 were *A. niger*. They validated the result of the loss of FB_2_ production in pattern 5 through a gene expression study. It was suggested that the loss of FB_2_ production in these strains was due to structural or regulatory mutations that changed gene expression. Conversely, a total of 18 *A. welwitschiae* isolates from onions in Saudi Arabia carrying all the FB_2_ *fum* genes were confirmed to produce FB_2_ (Gherbawy et al., 2015). In this study, we identified 22 *A. niger* strains with two or more *fum* genes. A total of 21 of the *A. niger* strains in our study are similar to pattern 3 of Palumbo et al. (2013). Furthermore, we found a new pattern with isolate 131-2A in which only the *fum13* was not amplified (**Figure 4A**). A gene expression study of the *fum* genes will assist in the validation of their FB_2_ production. The amplification and presence of *fum* genes were noted as biomarkers for FB_2_ production in *A*. section *Nigri* (Massi et al., 2016b).

OTA is a secondary metabolite produced by *Aspergillus* and *Penicillium* species. It is the most toxigenic ochratoxin detected in food in several countries (Gil-Serna et al., 2019; Sartori et al., 2010). It has been reported in various foods such as grapes, spices, grains, coffee beans, and nuts (Bayman et al., 2002; Massi et al., 2016b, a). In total, 32% of *A. niger* strains isolated from Brazilian nuts, coffee beans, grapes, cocoa, and onions produce OTA, whereas 74% of these *A. niger* strains were FB_2_ producers (Massi et al., 2016a). A multiplex PCR was used to detect the essential genes, *pks* and *radH*flavin-dependent halogenase (*radH*), involved in OTA production in the genome of *A. niger* and *A. welwitschiae*; however, 95.2% of the OTA-nonproducing *A. niger* and *A. welwitschiae* do not possess these genes. The loss of OTA production in these strains was attributed to gene deletions in the OTA biosynthetic gene cluster (Massi et al., 2016a). PCR assays have been developed and used for the early detection of OTA-producing *Aspergillus* species (Patiño et al., 2005). Ferracin et al. (2012) investigated 119 isolates of *A. niger* from dry fruits, Brazil nuts, and coffee beans for their OTA production using *pks* genes based on the nucleotide sequence of *A. niger* strain CBS 513.88, and 26% of these strains were reported to produce OTA. The primer pair PKS15KS (776 bp) successfully amplified a single band in the OTA-producing strains. Conversely, this band was not detected in the OTA-nonproducing strains (Ferracin et al., 2012). This result is similar to our results shown in **Figure 5**. Although only 1.5% of the *A*. *niger* isolates in our study were positive for *pks15ks*, indicating OTA production in these strains, similar studies have proven that there are lower percentages of OTA-producing *A. niger* strains detected in food samples when compared to FB_2_ (Ferracin et al., 2012; Massi et al., 2016a; Susca et al., 2016). Furthermore, onions contain various bioactive compounds and have antifungal properties (Sagar et al., 2022). These antifungal compounds could result in low contamination with mycotoxic fungal agents.

## 5 Conclusion

Recently, attention has been paid to the detection of mycotoxigenic *A. niger* strains isolated from vegetable and spice products to ensure better human health. Onion products in West Africa must be certified for their mycotoxin contamination level before acceptance for export to encourage onion production in this region. To the best of our knowledge, this is the first reported case of FB_2_ and OTA-producing *A*. section *Nigri* being isolated from onion bulbs in Nigeria. Therefore, through its Agricultural Research Institutes and Services, Nigeria is now paying more attention to the mass production of quality onion seedlings for health, food security, and medicine. A quick intervention using multiplex PCR for the early detection of mycotoxins such as FB_2_ and OTA-producing *A*. section *Nigri* that mostly affect onion products will help to achieve quality planting materials, large-scale production, and exportable onions in Nigeria and sub-Saharan Africa.

## Data Availability

The raw data supporting the conclusions of this article will be made available by the authors, without undue reservation.
