# Supplementary material for: Fumonisin and ochratoxin-producing strains of Aspergillus section Nigri are associated with onion (Allium cepa L.) bulbs sold in markets in southwest Nigeria
Source: Front Fungal Biol. 2025 Mar 24;6:1563824. doi: 10.3389/ffunb.2025.1563824 (PMC12056510; doi:10.3389/ffunb.2025.1563824)
Supplement: Supplementary file 1 [file DataSheet1.zip › Supplementary figure captions.docx]

**Supplementary Figure S1**

The agarose gel showing the fumonisin biosynthetic gene *fum*1 (452 bp) detected in the *A*. *niger* isolates as displayed in Figure 3A.

**Supplementary Figure S2**

The agarose gel showing the fumonisin biosynthetic gene (*fum*1) as displayed in Figure 3B.

**Supplementary Figure S3**

The agarose gel showing the multiplex PCR, primer sets A, and primer sets B of fumonisin biosynthetic genes detected in some of the *A. niger* isolates as displayed in Figure 4A.

**Supplementary Figure S4**

The agarose gel showing the multiplex PCR, primer sets A, and primer sets B of fumonisin biosynthetic genes detected in some of the *A. niger* isolates as displayed in Figure 4B.

**Supplementary Figure S5**

The agarose gel showing the multiplex PCR, primer sets A, and primer sets B of fumonisin biosynthetic genes detected in some of the *A. niger* isolates as displayed in Figure 4C.

**Supplementary Figure S6**

The agarose gel showing the ochratoxin (OTA) biosynthetic gene *pks15k*s (766 bp) detected in some of the *A. niger* isolates as displayed in Figure 5.
