# Supplementary figures and images for: Fumonisin and ochratoxin-producing strains of Aspergillus section Nigri are associated with onion (Allium cepa L.) bulbs sold in markets in southwest Nigeria
Source: Front Fungal Biol. 2025 Mar 24;6:1563824. doi: 10.3389/ffunb.2025.1563824 (PMC12056510; doi:10.3389/ffunb.2025.1563824)

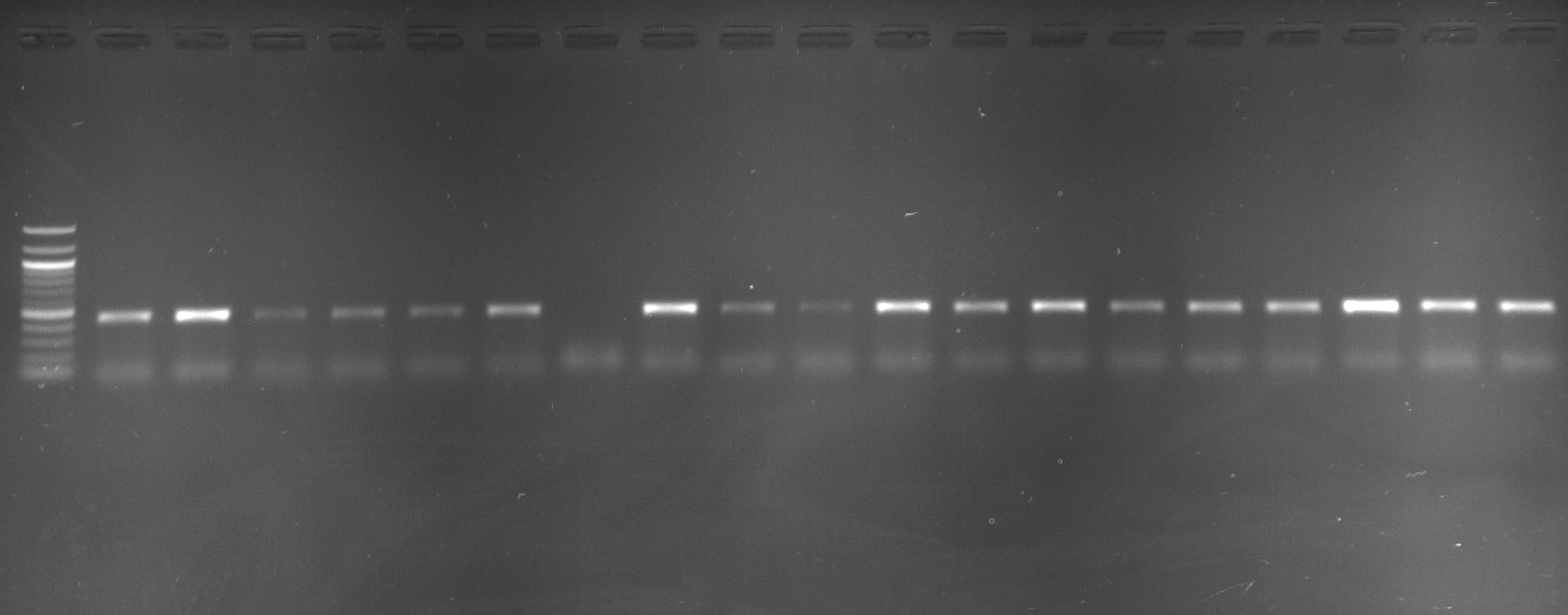

Supplement: Supplementary file 1 [file DataSheet1.zip › Supplementary Figure S1.JPEG]

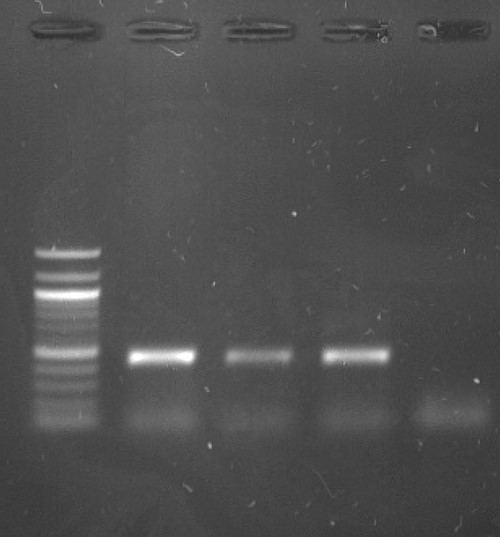

Supplement: Supplementary file 1 [file DataSheet1.zip › Supplementary Figure S2.JPEG]

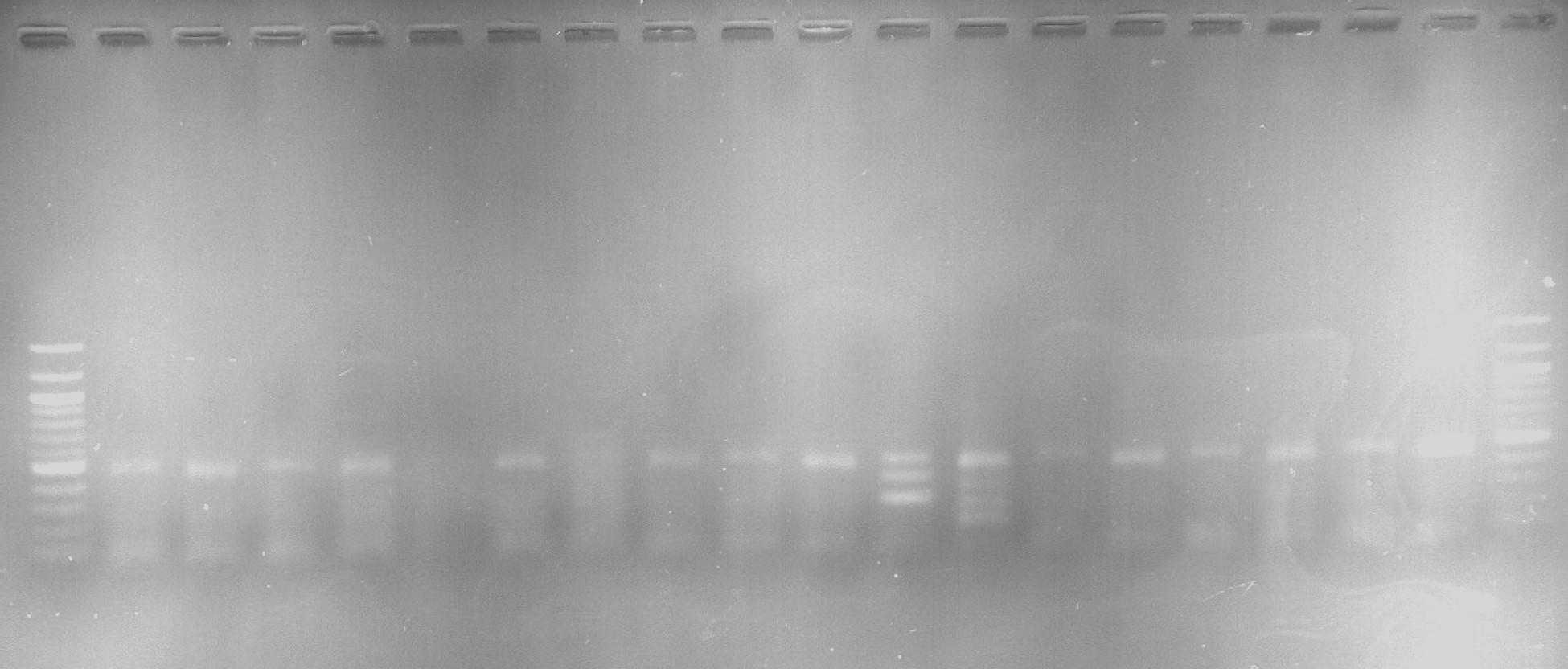

Supplement: Supplementary file 1 [file DataSheet1.zip › Supplementary Figure S3.JPEG]

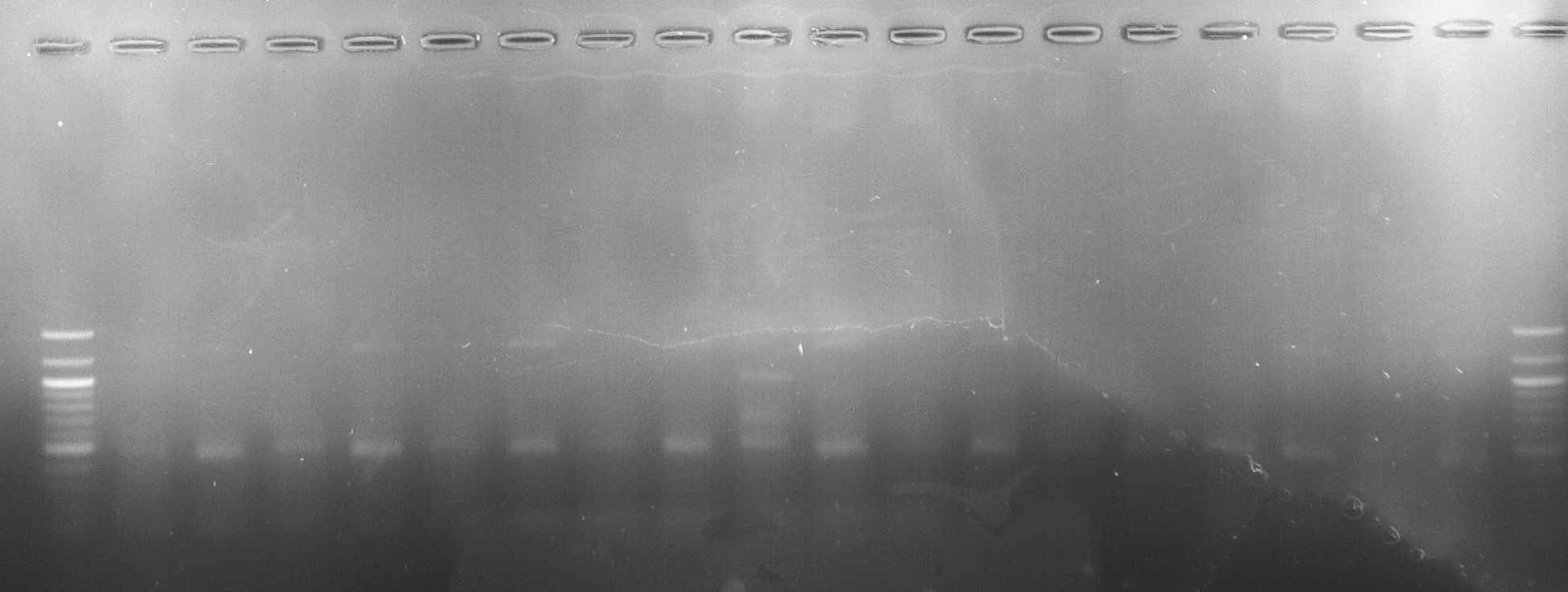

Supplement: Supplementary file 1 [file DataSheet1.zip › Supplementary Figure S4.JPEG]

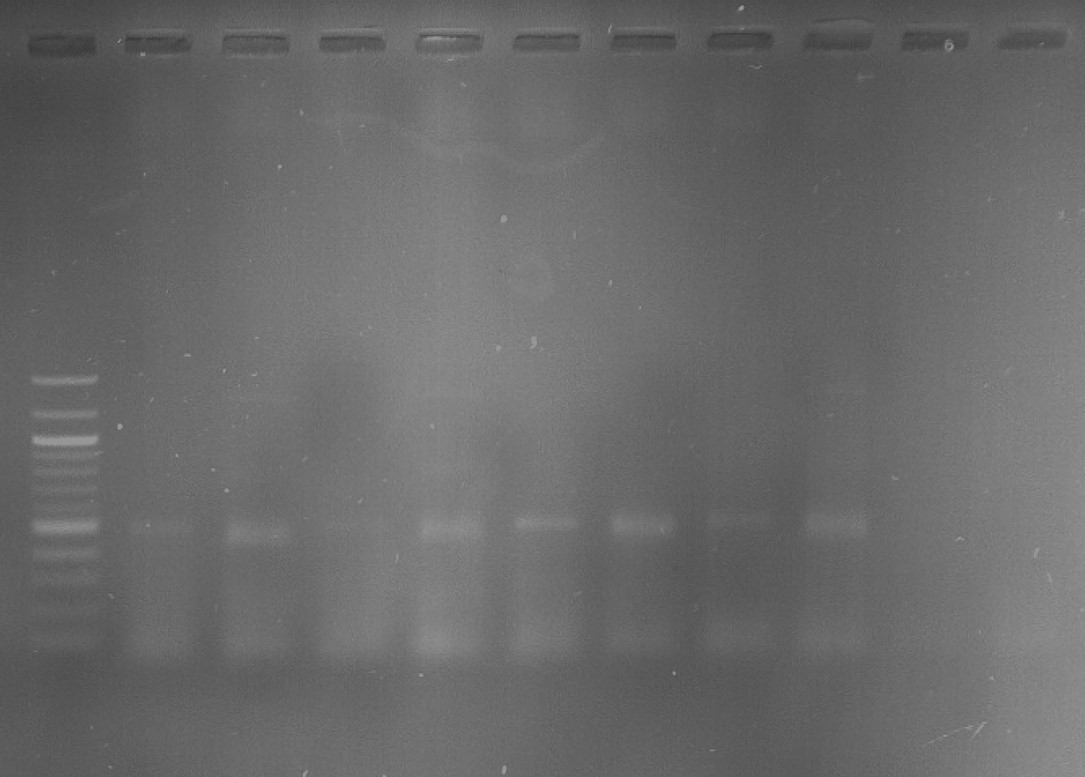

Supplement: Supplementary file 1 [file DataSheet1.zip › Supplementary Figure S5.JPEG]

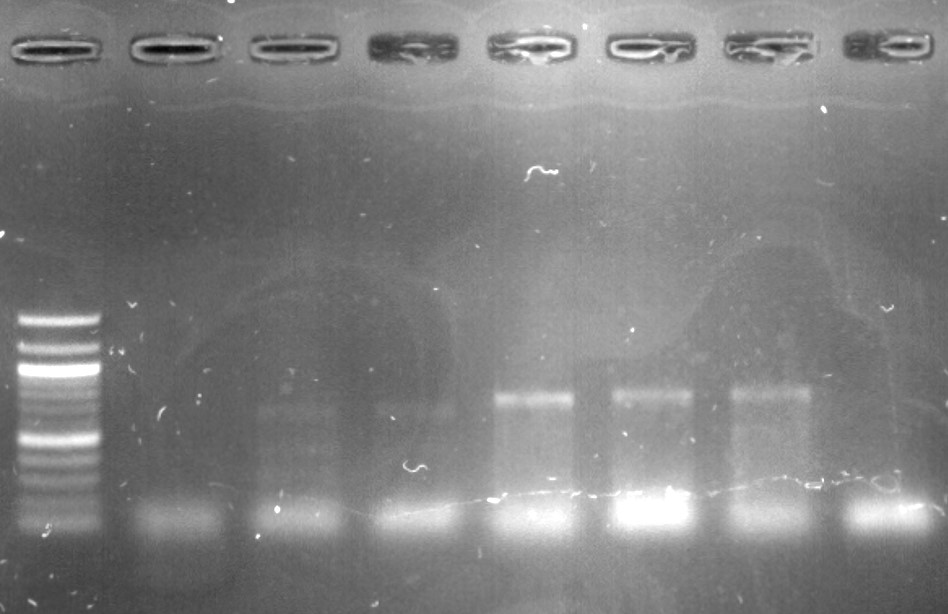

Supplement: Supplementary file 1 [file DataSheet1.zip › Supplementary Figure S6.JPEG]
